# Religious paternalism and racial prejudice in obstetric surgery in Mexico

**DOI:** 10.1590/S0104-59702024000100069

**Published:** 2024-12-16

**Authors:** Bonnie Adorno Lucero

**Affiliations:** i Neville G. Penrose Chair in Latin American Studies and History/College of Liberal Arts/Texas Christian University. Fort Worth – TX – USA b.lucero@tcu.edu

A significant and timely intervention in the burgeoning field of Latin American reproductive histories, *Surgery and salvation* examines the relationship between surgical politics and reproductive governance in Mexico between the mid-eighteenth and early twentieth centuries. It is precisely during this period that Elizabeth O’Brien situates the origins of some of today’s most urgent reproductive injustices, including the spiritual, legal, and medical prioritization of the unborn over women, the stark and persisting racial disparities in maternal and infant health, and the ongoing pattern of obstetric violence that disproportionately affects poor and racially marginalized women.

O’Brien traces the historical roots of those enduring problems to eighteenth-century theological debates, which originated doctrinal innovations pitting the spiritual salvation of the unborn against the corporeal survival of the mother. The radical reconceptualization of personhood legitimized, and even demanded, obstetric violence, first institutionalized in the religious imperative to perform cesarean operations to rescue even the smallest products of conception from eternal damnation. Such paternalistic discourses of salvation proved crucial for legitimizing exploratory and experimental obstetric procedures, often more interested in producing surgical knowledge than curing patients. Impoverished and racially marginalized women bore the brunt of the corporeal sacrifice demanded in the name of surgical advancement, carving deep disparities throughout reproductive medicine. Indeed, O’Brien (2023, p.16) demonstrates that surgery emerged as a “fault line demarcating … embodied prejudice.” Paternalistic discourses of salvation evolved alongside political and medical exigencies, but racial and class prejudice remained central, perpetuating over a century of reproductive injustice in Mexico.

The profound injustices perpetrated under the banner of salvation are carefully laid out across five chronological sections. The first four provide the core periodization defined by specific iterations of salvational discourse: religious (1745-1840), post-colonial (1840-1876), positivist (1876-1911), and revolutionary (1911-1940). Each of these sections, which studies a distinct set of surgeries, is comprised of two chapters – one examining the theoretical discussions of reproductive surgery during the period, and the second exploring the ways these theories manifested in policy and clinical practice. Together, these chapters show how both religious paternalism and racial prejudice persisted at the very heart of obstetric surgery.

Foundational to the book’s argument is O’Brien’s careful excavation of the theological origins of fetal personhood. In chapter one, she painstakingly unpacks the cultural context, logics, and far-reaching implications of Italian priest Francesco Emanuele Cangiamila’s 1745 *Sacred embryology*, which posited that ensoulment occurred early in pregnancy. Part of a broader corpus of theological embryology, Cangiamila helped catalyze a dramatic expansion in Catholic notions of personhood, effectively arguing for the individuation of the ensouled fetus from its mother. This emergent metaphysical paradigm rendered conditional baptism of the unborn (by way of transvaginal injection of holy water, for instance) unsatisfactory, demanding that products of conception “as small as a grain of barley” be extracted from the womb to properly receive the sacrament. Cesarean surgery was, thus, deemed necessary to “perfect” baptism.

O’Brien reveals how the zealous pursuit of perfecting the sacrament hinged on religious paternalism and patriarchal control. A cornerstone in this theological project was the gendering of the ensouled fetus as male, theoretically even in the very early stages of pregnancy prior to the formation of sex organs. Personhood thus became explicitly gendered, with the potential existence of a “male” fetus, regardless of its state of development, rendered more significant than an actual living human woman. The theological prioritization of baptizing the unborn vested priests with extreme power over women’s spiritual lives and corporeal fate. Under constant suspicion of being pregnant, women became subject to spiritual coercion, with priests being encouraged to ascertain gestational status even if it required betraying the sanctity of confession or engaging in gossip to expose a woman’s sexual conduct and to withhold absolution to obtain pre-emptive permission to operate.

That such spiritual blackmail proved absolutely central to the production of medical and surgical knowledge raises important questions about how these theological debates about fetal personhood inflected the evolving legal and institutional landscapes of late colonial Spanish America. For instance, how does our understanding of the dramatic expansion in the late-eighteenth and early-nineteenth centuries of religious establishments for unwanted infants (*expósitos*) and unwed pregnant women, most of which employed medical personnel, change when we consider the mobilization of faith to gain medical access to vulnerable bodies? How did those institutions, wedded as they were to colonial concerns of public reputation, negotiate their obligations to uphold the secrecy of women’s sexual conduct against a theological discourse that allowed and, indeed, demanded its exposure at any cost to assure the spiritual salvation of the unborn?

The implications for legal debates on fertility control are equally as significant. The imperative of salvational cesarean placed women under constant suspicion of not doing enough to ensure the delivery of a healthy (male) child. Such a religious framework of maternal culpability offers a crucial cultural backdrop for mid-nineteenth-century legal discussions of infanticide by omission – a controversial juridical category whereby a woman could be held criminally responsible for failing to prevent a miscarriage, stillbirth, difficult labor, neonatal death, or any other unfavorable pregnancy outcome, even if preventing it was often quite impossible. This consideration of legal factors makes the powerful and wide-ranging implications of O’Brien’s argument clear: she lays bare the theological groundwork that enabled poor and racialized women to be criminalized for pursuing the same or similar surgeries that obstetricians were praised for, often in violation of women’s bodily integrity and corporeal survival. And this religious justification for differential access to and control over women’s bodies proved crucial in the production of medical and anatomical knowledge, which hinged on the medical commodification of marginalized women’s bodies in political contexts ranging from the subjugation of indigenous people of the late eighteenth century to national eugenic projects of the mid-1900s.

Salvational cesarean became a mandate across the Spanish Empire in the second half of the eighteenth century, as Few, Tortorici and Warren (2020), Rigau-Pérez (1995), and others have shown. But New Spain proved fertile terrain for its radicalization. Backed by formal mandates, cesarean surgery became a political and cultural weapon on New Spain’s northern frontier, as chapter two provocatively demonstrates. For priests, this form of surgery became a means to prove loyalty to the Crown through coercive evangelization; for indigenous women hailing from unconquered civilizations, it proved a deadly form of cultural effacement and colonial subjugation. Indeed, through analyses of a database of baptismal records compiled from Spanish missions, O’Brien compellingly posits that frontier priests further radicalized European theological interpretations of fetal personhood, operating not only on deceased women, but also on live ones.

While salvational cesarean entered New Spain’s frontier zone at a particularly crucial time of colonial warfare and race-making, paternalist discourses of salvation remained central to subsequent surgical knowledge production. For instance, O’Brien shows, in part two, how the medicalization of hysteria facilitated surgical access to women’s reproductive bodies in the mid-nineteenth-century post-colonial era. Physicians invoked hysteria to declare certain pregnancies “unnatural” due to the woman’s emotional state, social circumstances, physical disabilities, or illness. Pathologizing those pregnancies, in turn, transformed the women into imperiled victims in need of surgical salvation. In a context in which performing an abortion could result in losing the license to practice medicine, artificial premature birth enabled physicians to terminate pregnancies surgically by inducing preterm labor. Though these physicians did not necessarily envision the procedure as an abortion, O’Brien points out that the outcomes were generally not favorable to the survival of the fetus, or the mother for that matter.

Echoing Deirdre Cooper Owens’s pathbreaking study of enslaved women’s role in US gynecology (Owens, 2017), O’Brien exposes how poor and racialized Mexican women were consistently targeted for exploratory and experimental obstetric surgeries, ones in which anatomical and medical knowledge production took priority over the therapeutic needs of the patient. A case in point is the discussion, in section three, of the intensification of overt racial prejudice in surgical interventions on pregnant women. O’Brien observes a shift during the Porfirian period (1876-1911) in medical perceptions of Mexican women’s pregnancies from natural to problematic and in need of surgical intervention. Obstetricians argued that dystocia, or difficulty giving birth, was widespread in Mexico due to the supposed biological inferiority of indigenous women, evinced by their small pelvises. This pelvic deficiency thesis led some physicians to prescribe either surgical intervention during labor (a precursor of contemporary episiotomies) or reproductive controls, including ovariotomy and pregnancy termination, depending on the case. In the context of scientific racism, in which character and intellect were believed to be hereditary and racially determined, changes to a person’s organs or systems were understood to have the power to improve behavior and influence evolution. As such, surgical interventions on women’s reproductive bodies were deemed necessary to ensure the racial and cultural progress of the Mexican population, thereby saving the nation. Access to women’s bodies was crucial to the production of this racial science, and the dramatic expansion in state surveillance of poor and marginalized communities facilitated the commodification of women’s bodies for medical experimentation. Sanitary police rounded up women labeled prostitutes into prison hospitals, and these prisoner-patients became live fodder for ambitious obstetricians who performed experimental and exploratory surgeries that became foundational to modern gynecology.

Marginalized women, whether indigenous, impoverished, or devoid of patriarchal protections, were also more vulnerable to the experimental sterilization procedures discussed in part four. Upending the conventional wisdom that Mexican eugenicists were not surgically interventionist, O’Brien shows that large numbers of Mexican women did face eugenic sterilization. The types of surgical sterilization Mexican physicians employed depended on eugenic assessments. Permanent sterilization by way of tubal ligation was the option chosen for women with supposedly deficient pelvises to prevent them from giving birth. Alternatively, there was a theoretically more temporary procedure, known as vaginal bifurcation. This was a unique operation Mexican obstetricians used to reconcile (however ironically) Catholic prohibitions on bodily mutilation with eugenic sterilization, by surgically creating a second vaginal canal close to the cervix to suspend childbearing in women deemed racially desirable but temporarily unfit due to illness or social circumstances.

What all these sections so vividly capture is that the supposed moral quality of surgical procedures has historically been fluid and situational. The same procedure could be deemed immoral and unallowable in some circumstances, and morally imperative and beneficial in others. And the predictable but still demoralizing difference usually hinged on the dismissal of women’s experience, agency, and designs on their own lives and bodies. That a woman’s request for sterilization, birth control, or pregnancy termination most often proved insufficient – and in fact it still does in many contexts – underscores the enduring power of paternalist discourses of salvation.

With this book, O’Brien offers compelling answers to some of the most pressing questions confronting the field of reproductive history and opens new avenues of investigation about the relationship between theology and secular society. She rightfully re-centers Latin America, and Mexico, as a vanguard in the application and radicalization of theological ideas about fetal personhood, reproductive surgery, and gendered racialization. *Surgery and salvation* is absolutely essential reading for students of the Americas and for all those interested in women, reproduction, race, religion, and medicine – past and present.
